# Spontaneous Remission of Late Relapsing Membranous Nephropathy After Previous Immunosuppressant‐Induced Remission: Case Report

**DOI:** 10.1155/crin/6703642

**Published:** 2026-04-12

**Authors:** Yangming Cao

**Affiliations:** ^1^ Department of Internal Medicine, Division of Nephrology, UCSF Fresno, Fresno, California, 93701, USA; ^2^ The Nephrology Group, 568 E Herndon Ave Suite 201, Fresno, California, 93720, USA

**Keywords:** case report, late relapse, membranous nephropathy, Phospholipase A2 receptor mediated, spontaneous remission

## Abstract

This is the first detailed report of spontaneous remission of late relapsing membranous nephropathy (MN) after previous immunosuppressant‐induced remission. In March 2004, a 52‐year‐old Hispanic male presented with severe nephrotic syndrome with proteinuria of 13 g/24 h. The workup for infectious and autoimmune diseases was unrevealing. Renal biopsy showed MN (Stage II). A CT scan showed no malignancy but left pulmonary embolism and left renal vein thrombosis. Anticoagulation was started for asymptomatic thromboembolism. He achieved sustained complete remission of nephrotic syndrome after a prolonged, complicated treatment course of multiple immunosuppressants (corticosteroids and mycophenolate mofetil for 1 year, followed by alternating monthly corticosteroids and chlorambucil for 6 months, and again corticosteroids and mycophenolate mofetil for 6 years). Then in July 2021 (now at the age of 69 years), he developed nephrotic syndrome again with proteinuria of 6 g/24 h. Serological work‐up was all negative except for the elevated serum Antiphospholipase A2 receptor (PLA2R) antibody at 42 RU/mL (nl < 14). Renal biopsy in November 2021 showed again MN (Stage III to IV). Immunohistochemistry staining of the biopsy tissue for PLA2R1 antigen was positive. A decision was made to observe him instead of starting immunosuppressants. He was treated with diuretics and Lisinopril. Anti‐PLA2R antibody steadily improved to normal range and the nephrotic syndrome gradually improved and finally resolved by August 2023. There was no recurrence of nephrotic syndrome up to the most recent visit in March 2026. A review of the literature shows that a repeat biopsy in patients already known to have MN (especially those PLA2R‐mediated cases) with relapse of nephrosis and stable renal function may not be necessary. In routine practice, the watchful waiting strategy for relapse of MN is less often taken as it should be. Therefore, more patients with relapsing MN deserve an observation period before starting immunosuppressants. Hopefully, KDIGO guidelines can include this point for relapsing MN.

## 1. Background

Relapses of primary membranous nephropathy (MN) after 5 years of sustained complete or partial remission (CR or PR), the so‐called late relapses, were considered rare events [1, 2]. However, others reported a higher incidence [3, 4]. This is to present the first detailed case report of spontaneous remission of late relapsing MN after previous immunosuppressant (IS)‐induced remission. Only one similar milder case, without specific attention to clinical course, can be clearly identified in the literature [1]. Spontaneous remission of MN can occur in both initial episode and relapsing episode of nephrotic syndrome [2, 5]. However, a review of the literature suggests that the watchful waiting strategy for relapse of MN is less often taken than it should be [1, 4, 6–9]. To make it worse, the current Kidney Disease: Improving Global Outcomes (KDIGO) guidelines do not mention the watchful waiting strategy at all [10]. Instead, it directly gives instruction for ISs for initial relapse after therapy [10]. In addition, a repeat biopsy in the majority of patients already known to have MN with relapse of nephrosis and stable renal function may not be necessary [1].

## 2. Case Presentation

In March 2004, a 52 year‐old Hispanic male presented with bilateral leg edema for about one year. He was seen by a nephrologist for nephrotic syndrome with proteinuria of 13.0 g/24 h. Hepatitis B and C were ruled out. Renal biopsy showed MN (stage II). He was treated with prednisone 20 mg p.o. (orally) tid for 2 months, followed by 40 mg p.o. qd. In April 2004, his proteinuria was 9.3 g/24 h. His renal function was normal with serum creatinine of 0.9 mg/dL. Complete blood count was normal except the elevated white blood cell of 16.8 × 10^3^/μL which was likely due to prednisone. Liver function test was normal except serum albumin being low at 1.2 mg/dL. Lipid profile showed severe hyperlipidemia (total cholesterol 444 mg/dL, low‐density lipoprotein 308 mg/dL and triglyceride 291 mg/dL).

Then in late May 2004, the patient was seen by me for a second opinion. He had lost 40 pounds of body weight over the past 2 months despite good appetite. He ascribed the weight loss to the medications and so he stopped quinapril and diuretics. He also developed severe depression. The medicines he was still taking were prednisone (40 mg p.o. qd), lansoprazole, atorvastatin and alprazolam. Physical exam showed a blood pressure of 121/75 mmHg, a body weight of 81 kg and 2+ leg edema. Urinalysis showed 4+ protein, moderate blood, with a few granular and hyaline casts. He was started on furosemide, quinapril, calcium carbonate and sertraline, while continuing to take all current medicines except alprazolam. He was told that he might need combination immunosuppression in the near future. Due to MN and significant weight loss, malignancy should be ruled out. Further tests were ordered and he was to return in 3 weeks. Stool test was negative for occult blood and serum prostate specific antigen (PSA) was normal. His blood test was negative for HIV, RPR and ANA with normal complements (C3 and C4). Lower extremity ultrasound showed no deep venous thrombosis. In early June 2004, CT of chest, abdomen and pelvis without and with intravenous contrast showed no malignancy but intraluminal filling defects within the descending branch of left pulmonary artery compatible with pulmonary emboli and non‐occlusive thrombus in the left renal vein. He was immediately called back and hospitalized for asymptomatic thromboembolism. He was started on subcutaneous enoxaparin followed by oral warfarin. Colonoscopy removed a small polyp which was pathologically tubular adenoma. In the office visit a few days after the hospital discharge, prednisone 40 mg p.o. qd was continued with addition of mycophenolate mofetil (1000 mg p.o. bid) and hydrochlorothiazide. Quinapril (dose later maximized), warfarin, atorvastatin and calcium carbonate were continued. In July 2004, proteinuria was better with random urine protein/creatinine ratio of 2.5 g/g and so prednisone was reduced to 20 mg p.o. qd. His proteinuria improved to 1.0 g/g by September 2004 when prednisone was reduced again to 10 mg p.o. qd. Afterwards, proteinuria fluctuated but gradually worsened.

By April 2005, proteinuria was worse to 2.3 g/g with serum albumin of 2.2 g/dL. In May 2005, the ISs (prednisone and mycophenolate mofetil) were escalated to the alternating monthly cycles of corticosteroids (intravenous methylprednisolone 1000 mg qd for 3 days followed by prednisone 40 mg (0.5 mg/kg) p.o. qd for 27 days) on month 1, 3, 5 and chlorambucil (14 mg, about 0.2 mg/kg) p.o. qd on month 2, 4, 6. His proteinuria gradually and steadily improved to 0.42 g/g by November 2005. However in early December 2005 (about 1 week after he completed the 6 months’ cyclical treatment of ISs), his proteinuria worsened to 3.9 g/g with serum albumin of 3.6 g/dL. He complained of fatigue and muscle ache all over the body. He was immediately started on prednisone 80 mg (1 mg/kg) p.o. qd and mycophenolate mofetil 500 mg p.o. bid (which was raised to 1000 mg p.o. bid later). Valsartan was added to quinapril (the doses were both maximized later). The combination of angiotensin‐converting enzyme (ACE) inhibitors and angiotensin receptor blockers (ARBs), now obsolete, were then a common practice for treating proteinuria. Proteinuria improved to 1.1 g/g by February 2006. At that time, warfarin was discontinued because he complained of having bruise and the serum albumin was normal at 3.6 mg/dL. Prednisone was tapered but kept at low dose and finally discontinued in December 2006. Then proteinuria remained in the range of 0.2–0.9 g/g except one‐time value of 3.3 g/g in Oct 2008. Afterwards proteinuria fluctuated but remained less than 0.8 g/g. Mycophenolate mofetil was tapered and finally stopped in April 2012, by which time proteinuria was less than 0.2 g/g for more than one year.

From April 2012 to February 2014, proteinuria was about 0.1–0.4 g/g and microalbuminuria 16–152 mg/g. Serum creatinine remained normal at 0.8 mg/dL and urinalysis was normal until February 2014 when microalbuminuria suddenly worsened with random urine albumin/creatinine ratio of 2590 mg/g. This microalbuminuria value was likely erroneous because urinalysis showed only trace protein. His BP was normal and had no leg edema. Diuretics and valsartan were stopped but quinapril was continued. He was scheduled to come back in 1 month to recheck. However he was lost to follow up. There was (now retrospectively) a proteinuria of 0.28 g/g in 2016 per primary care physician’s records.

In July 2021 (now at the age of 69 years), he saw his primary care physician for leg edema, fatigue and dizziness. He was found to have relapsing nephrotic syndrome, with normal renal function (serum creatinine 0.68 mg/dL) and hypoalbuminemia (serum albumin 3.0 g/dL). Urinalysis showed 4+ protein and no blood with proteinuria of 6 g/24 h. He also had hyperlipidemia. In October 2021, he was seen by a nephrologist for consultation. Serological work‐up (hepatitis B and C, anti‐nuclear, anti‐DsDNA, anti‐glomerular basement membrane and anti‐neutrophil cytoplasmic antibodies) were all negative, except for the elevated anti‐phospholipase A2 receptor (PLA2R) antibody at 42 RU/mL (nl < 14) and mild elevated rheumatoid factor at 16 IU/mL (nl < 14). Renal biopsy in November 2021 again showed MN (stage III to IV), focal segmental glomerulosclerosis (which was likely related to MN), acute tubular injury, severe arteriosclerosis, and mild (10%) interstitial fibrosis and tubular atrophy. Immunohistochemistry staining for PLA2R1 antigen was positive in glomerular capillary wall deposits while the staining for Thrombospondin T1D7A antigen (THSD7A) was negative. The proteinuria was worse to 7.63 g/g in December 2021 and so the patient was recommended to start ISs. However the patient came to me for a second opinion and was seen in February 2022. On exam, his blood pressure was 144/80 mmHg with 1+ leg edema. A decision was made to observe him instead of starting ISs. He was started on diuretics, potassium chloride and continued on amlodipine. The dose of lisinopril was raised to 40 mg p.o. qd. Colonoscopy was done with one polyp removed and PSA was normal. With anti‐PLA2R antibody steadily improving to normal range, his nephrotic syndrome gradually improved and finally resolved by August 2023 (Table 1). His renal function remained stable during this whole period. On last visit (March 2026), he was doing well with normal blood pressure and without leg edema. His medicines were furosemide 20 mg p.o. bid, potassium chloride 10 meq p.o. bid, lisinopril 40 mg p.o. bid and vitamin D.

**TABLE 1:** Laboratory tests of the late relapse episode of membranous nephropathy over time.

	10/2021	3/2022	5/2022	8/2022	10/2022	8/2023	8/2024	2/2026
Serum Cr (mg/dL)		0.79	0.82	0.94	0.9	0.78	0.83	0.82
Serum albumin (g/dL)		3.4	3.6	3.7	3.7	3.7	4.2	3.8
Urine protein/Cr ratio (g/g)	7.63	2.48	1.18	0.56	0.60	0.16	0.06	0.08
Urine albumin/Cr (mg/g)			866	323				
Urine RBC/HPF	0	0	0	0	0	0	0	0
Serum anti‐PLA2R antibody (RU/mL)^∗^	42	14	8	10	10		< 4	

*Note:* Cr = creatinine.

Abbreviations: HPF = high power field; RBC = red blood cell.

^∗^Normal range for anti‐PLA2R antibody is < 14 RU/mL.

## 3. Discussion

Relapse of primary MN typically occurs within the first few years of achieving remission [1]. Literature review revealed only six studies on late relapse (i.e., relapse beyond 5 years after remission) in primary MN, as summarized in Table 2 [1–4, 8, 11]. Although late relapse MN was considered rare at 1%–6% [1, 2], others reported a much higher relapse rate [3, 4]. A cumulative incidence (CI) of relapse of 27% at 5 years and 37% at 10 years was reported [3]. An even higher CI of 40% was reported at 15 years [4]. One should be cautious in interpreting the relapse rate or CI of relapse. Some studies counted relapse among total patients [1], while others counted relapse among only patients who had achieved remission [2–4]. Since fewer patients are followed beyond 5 years, the confidence interval for the estimate of cumulative incidence (CI) is wide [3]. Nevertheless, these studies showed that there are increased chances of relapse as time passes. Therefore how to properly manage the late relapse is becoming more important. Similar to the treatment of MN relapse in general (see below), a majority of patients with late relapse in the literature were treated with ISs without given an observation period (Table 2) [1, 4, 8, 11].

**TABLE 2:** Summary of late relapse of membranous nephropathy in the literature.

	Initial MN		Late relapse		
	IS^∗^	CR or PR^∗^	RR or CI^∗^	IS^∗^	CR or PR^∗^
van den Brand et al. (2014)[3]	48.9% (124 of 254 patients) received IS.	83.4% (206 of 247, with 7 patient no data) had CR or PR	22.3% (46 of 206) had relapse CI: 27% at 5 years; 37% at 10 years	Not reported	54% (25 of 46) had PR

Kanigicherla et al. (2016) Ref. [4]	128 patients (67 patients spontaneous PR; 33 patients had IS induced PR, total 100 patients)	78.1% (100 of 128) had PR	31% (31 of 100) had relapse CI: 19% at 5 years; 40% at 15 years	90% (28 of 31) had IS 3 patients ineligible due to co‐morbilities	82% (23 of 28) had PR. The outcome of the 3 patients not reported

Polanco et al. (2010) [2]	Spontaneous remission occurred in 32% (104 of 328 patients)	CR or PR	5.7% (6 of 104)	4 patients not received IS; 2 patients received IS	All 4 patients without IS and 1 with IS got into remission; the other one with IS had no remission

Barnes et al. (2011) [11]	3 patients (3 of 7 patients met criteria of late relapse) and all these 3 patients received IS	CR or PR	Unknown	All received IS	Not reported

Peleg et al. (2021) Total 16 patients Ref. [1]	IS 12 patients	CR or PR		IS	CR or PR
	No IS 1 patient (No. 7)	CR		IS	PR
	IS 1 patient (No. 8)	PR		IS	No
	No IS 1 patient (No. 10)	CR		No IS	No
	IS 1 patient (No. 15)	CR		No IS	PR

Total 16 patients	87.5% (14/16) received IS	87.5% (14/16)	Appx. 1% (16 of about 2000 patients)	87.5% (14/16) received IS	87.5% (14/16)

Kumar et al. (2023) Ref. [8]	100% (5 of 5 patients) all received IS	100% (5 of 5)	Unknown	100% (5 of 5) all received IS	100% (5 of 5) had CR or PR

^∗^CI = cumulative incidence; CR = complete remission; IS = immunosuppressants; PR = partial remission; RR = relapse rate.

A large‐volume center (Columbia University, USA) that sees approximately 100 patients with MN each year found only 16 such cases in their database over a 20‐year span (from 1999 to 2019), which translated into approximately 1% of their MN cohort (Table 2) [1]. All of the 16 patients achieved CR or PR of the initial MN, 14 of whom received IS, while 2 did not (both of these 2 patients achieved CR). For the late relapse of MN, there were also 14 patients receiving IS and 2 did not. Patients had favorable long‐term renal outcomes over a median 21‐year follow‐up period, with 14 of the 16 patients (87.5%) in CR or PR at study conclusion. Of the 2 patients who did not receive IS for the initial MN but achieved CR, one patient went into PR after receiving IS for the late relapse, while the other patient did not receive IS and did not achieve remission. Only one of the 14 patients who received IS for the initial MN was not treated with IS for the late relapse (Case No. 15 of their series). This patient had proteinuria of 7.0 g/g with the initial MN, and he achieved CR after receiving corticosteroids. On the late relapse, his proteinuria was 4.9 g/g, followed by going into PR without IS (Table 2) [1].

In comparison, our patient had more severe nephrotic syndrome complicated by thromboembolism with the initial MN and achieved CR after a prolonged, complicated treatment course of multiple IS (corticosteroids and mycophenolate mofetil for 1 year, followed by alternating monthly corticosteroids and chlorambucil for 6 months, and again corticosteroids and mycophenolate mofetil for 6 years). He developed the late relapse about 10.5 years after he achieved sustained CR of initial MN. The reasons an observation approach for the relapse was chosen were three‐fold: (1) clinical symptom was not severe with only leg edema and a serum albumin of 3.0 g/dL despite that his proteinuria was worse to 7.6 g/g; (2) anti‐PLA2R antibody (when first checked at 3 months after his presentation) was not very high (at 42 IU/mL); and (3) renal biopsy showed “late stage” of MN (Stage III‐IV), although the pathological staging of MN was generally considered not associated with the prognosis. Fortunately, he attained CR of the relapsing MN without IS. Over the next 2.5 years, there was no further relapse. Only one similar milder case (i.e., IS‐induced remission of initial MN, with late relapse followed by spontaneous remission), which was in a collective report of case series and thus lacked clinical details, can be clearly identified in the literature as mentioned in the above paragraph (Table 2) [1]. This patient, similar to our patient, also had positive serum anti‐PLA2R antibody.

Spontaneous remission is a well‐known characteristic of primary MN [2]. An observation approach is taken for appropriately selected patients after risk stratification (KDIGO guidelines 2021: Practice Point 3.2.1 and Practice Point 3.3.1 to Practice Point 3.3.3) [10]. However, for the relapsing MN, KDIGO guidelines directly give instruction for IS (though it may be a different IS regimen) for initial relapse without mentioning an observation period (Practice Point 3.4.1) [10]. UpToDate suggests a 6‐month observation period with general supportive measures to reassess the risk for progressive disease, using the same approach as that for newly diagnosed patients with primary MN, even if the patients previously received IS for their initial presentation [12].

How well is this observation (i.e., watchful waiting) strategy followed in routine practice? Unfortunately, not well. A prospective cohort of 103 consecutive Japanese patients with primary MN were treated with IS for the initial episode of nephrotic syndrome between 1988 and 2005 [6]. Additional IS therapies were considered for patients who had relapsed to heavy proteinuria over 2 months. During the observation over an average of 4.8 years, 40 relapses occurred in 30 patients, of which 32 relapses in 22 patients were treated with additional IS (only 8 relapses in 8 patients were not treated). Relapses occurred frequently but were generally benign in severity. The authors noticed the effects of retreatment for relapses were obtained more quickly and definitively compared with those achieved with initial therapy. All, except one, of the retreated relapse cases achieved remission after an average of 5.1 months, and the remission rate for all relapses was 94.8% at 2 years. However, the cumulative cyclophosphamide dosage of patients with additional therapy exceeded 10 g. The outcome of the 8 relapses that were not treated was not specifically reported.

In a retrospective analysis in Czech Republic for the period of 1991–2002, of the 45 patients who developed either a CR or a PR with or without initial IS, 14 relapsed to nephrotic range proteinuria. Another round of IS was offered to all 14 patients and was given to those who accepted the treatment [7]. A UK center retrospectively analyzed the adult patients diagnosed with primary MN between 1980 and 2010. Thirty‐one of 100 patients who achieved PR had at least one relapse of nephrotic proteinuria during the follow‐up period, some of whom were apparently late relapse cases. Immunosuppressive therapy was given to all patients for relapse except 3 patients who were deemed ineligible in view of comorbidities [4]. Similarly, a retrospective study from a hospital in northern India identified 5 patients with MN who presented with relapse of proteinuria from January 2021 to December 2021 after five years of remission. All of the 5 cases of late relapses were treated with IS (3 of which had had worsening renal function) [8].

The above studies from different countries spanning a total of about 40 years (from 1980 to 2019) suggest that the watchful waiting strategy for relapse of MN is less often taken than it should be [1, 4, 6–9, 11]. This is in contrast to an earlier report which showed about half of the patients with relapse of nephrotic syndrome due to primary MN had a spontaneous remission, although it was unclear how many of these patients had received IS for their initial nephrotic syndrome [5]. Large cohort‐based risk stratification scores have allowed international consensus on initial treatment strategies of MN (KDIGO) [10]. Efforts should now be focused on relapsing patients to enhance the prediction of spontaneous remission probability and long‐term risks, enabling personalized evidence‐based treatment strategies [9]. Interestingly, in a prospective study of 7 primary MN patients who had relapse or resistant disease, all 4 patients with the finding of immune complex deposits on repeat renal biopsy received IS, while the other 3 patients whose biopsy showed that the deposits had been eradicated was given only conservative care [11]. They found that those 3 patients had very high intakes of salt and protein, based on 24‐h urine testing. Reducing their high salt intake sharply lowered proteinuria to the subnephrotic range, and serum creatinine stabilized [11].

Rates of relapse were not statistically different between the spontaneous remission group and the IS‐induced remission group [4]. None of the 11 clinical variables (including demographics and clinical parameters) at the time of achieving remission could be shown to predict subsequent development of relapse [4]. A historical cohort of patients treated with cyclophosphamide for 12 months (vs. 6 months) had a lower relapse rate [13]. However, one should weight the risk and benefit of treatment of longer duration [13]. Emerging genetic risk scores may play a role in risk stratifying patients for the likelihood of late relapse [1, 14]. Persistent positivity or reappearance of serum anti‐PLA2R antibody is highly predictive of relapse [9]. However, recurrence of PLA2R‐related MN was reported in the absence of serum anti‐PLA2R antibody reappearance. A group of 16 patients were found to be negative for serum anti‐PLA2R antibody by indirect immunofluorescence (IIF) and enzyme‐linked immunosorbent assay (ELISA) testing at clinical relapse of MN [9]. However, eight of the 16 patients had renal biopsy at the time of relapse, and PLA2R‐positive deposits were found in all of the 8 patients [9]. Fourteen of the 16 patients received IS for relapse.

Our patient presented with MN long before anti‐PLA2R antibody tests were available. However, it was found to be PLA2R related on late relapse of nephrotic syndrome many years later. Repeat renal biopsy again showed MN. In the case series of Columbia University, all 11 repeat biopsies in late relapse revealed MN [1]. Given that serum anti‐PLA2R antibodies can be followed to assess impending relapse or remission in cases when MN is PLA2R‐mediated, i.e., 70%–80% of the time, the authors questioned the utility of repeat biopsies in patients already known to have MN with relapse of nephrosis and stable renal function [1]. Instead, they would recommend pursuing a biopsy in patients with known history of MN if there are other clinical indications, other than relapse of nephrosis. These include unexpected decline in renal function, which might be due to transformation to crescentic glomerulonephritis or associated acute interstitial nephritis or acute tubular necrosis. Likewise, those with long‐standing diabetes and those with indications of a monoclonal disease might benefit from rebiopsy, especially if they are PLA2R‐negative or unknown for initial MN presentation [1].

## 4. Conclusions

Late relapses of MN may not be as rare as thought before. This is the first detailed report of spontaneous remission of late relapsing MN after previous IS‐induced remission. Only one similar patient, which had no detailed description, can be clearly identified in the literature. A repeat biopsy in patients already known to have MN (especially those PLA2R‐mediated cases) with relapse of nephrosis and stable renal function may not be necessary. In routine practice, the watchful waiting strategy for relapse of MN is less often taken than it should be. Therefore, more patients with relapsing MN deserve an observation period before starting ISs. Hopefully, KDIGO guidelines can include this point for relapsing MN.

**Nomenclature**
CI: Cumulative incidence
CR: Complete remission
IS: Immunosuppressants
MN: Membranous nephropathy
PLA2R: Phospholipase A2 receptor
PR: Partial remission

## Author Contributions

Yangming Cao is the sole author of the article and is fully responsible for its content.

## Funding

No funding was received for conducting this study.

## Ethics Statement

This case report is based on the review of medical records and is not required for ethic approval in this institution. And so, there is no need for the patient’s consent to participate.

## Consent

The patient gave written informed consent for publication of his clinical details. But there are no identifying images in this article.

## Conflicts of Interest

The author declares no conflicts of interest.

## Data Availability

Data sharing is not applicable to this article as no datasets were generated or analyzed during the current study.
